# Injury Incidence in Professional Football Is Highest During Mid- and Late-Season: A Three-Season Retrospective Cohort Study

**DOI:** 10.3390/jfmk11030333

**Published:** 2026-08-25

**Authors:** Mehmet Mesut Çelebi, Atilla Çağatay Sezik

**Affiliations:** 1 Department of Sport Medicine, Faculty of Medicine, Ankara University, Ankara 06590, Turkey; 2 Department of Physiotherapy and Rehabilitation, Faculty of Health Sciences, Yüksek İhtisas University, Ankara 06520, Turkey; cagataysezik@hotmail.com

**Keywords:** football, seasonal variation, sports injuries, time-loss

## Abstract

**Background:** Injuries are a major concern in professional football because they may reduce player availability and affect team performance. Injury risk may vary across different phases of the season in relation to changes in training and competition demands, recovery, and cumulative workload. **Objectives:** To describe the epidemiology of injuries in a professional football team over three consecutive seasons and to evaluate seasonal variations in injury characteristics and injury-related time-loss. **Methods:** This retrospective study analyzed 260 time-loss injuries sustained by 78 of 96 registered male first-team players (mean age 27.0 ± 4.5 years) from a professional football club in Türkiye over three consecutive seasons (2022–2023 to 2024–2025). Total team exposure was 33,609 player-hours (2409 match player-hours and 31,200 training player-hours). Injuries were classified according to injury setting, seasonal period, anatomical location, injury type, and injury-related time-loss. Exploratory generalized estimating equations (GEE) analysis accounted for repeated injuries within players. **Results:** A total of 260 injuries were recorded; 62.3% occurred during training and 37.7% during matches. The overall injury incidence was 7.7 per 1000 player-hours (95% CI, 6.8–8.7), with rates of 40.7 per 1000 match player-hours (95% CI, 33.0–49.6) and 5.2 per 1000 training player-hours (95% CI, 4.4–6.1). The groin–thigh–lower-leg region (52.7%) and the thigh (25.4%) were the most frequently affected anatomical sites, while muscle injuries accounted for 43.5% of all injuries. Estimated injury incidence was highest during the mid- and late-season (9.29 and 9.12 per 1000 player-hours, respectively) and lowest during the preparation period (2.55 per 1000 player-hours). Exploratory GEE analysis showed significant differences in injury counts across seasonal periods (Wald χ^2^ = 41.35, *p* < 0.001). Compared with the late season, injury incidence was lower during the preparation period (IRR 0.17, 95% CI 0.09–0.33; *p* < 0.001), whereas no significant differences were observed for the early or mid-season. **Conclusions:** Injury incidence was lowest during the preparation period and highest during the competitive season. Because exposure estimates were based on team-level rather than individual player-level data, these findings should be interpreted cautiously. Prospective multicentre studies with individual exposure data are needed.

## 1. Introduction

Football is one of the most widely played sports worldwide and, due to its high physical demands, carries a substantial risk of injury. High-intensity activities in football, such as sprinting, sudden changes in direction, acceleration, deceleration, jumping, and physical contact, make athletes susceptible to various injuries. Injuries in professional footballers can affect not only the health of the athletes but also team performance [1], match results, and the economic resources of the clubs. Ekstrand et al. have shown that a decrease in player availability in elite football teams negatively impacts team success [2].

Epidemiological studies that provide information about the frequency, type, anatomical location, and severity of football injuries play a key role in developing effective injury prevention strategies [3,4]. The frequency, types, and anatomical distribution of football injuries have been investigated for many years [5]. Previous studies have shown that the majority of injuries in professional football players occur in the lower extremities, with muscle injuries being among the most common injury types [2,6]. However, the distribution of injuries can be affected by many factors, such as team level, age group, training load, competition intensity, and seasonal periods.

Injury risk may vary across different phases of the season because of changes in training and competition loads, players’ physical readiness, and cumulative fatigue. Training and competition loads are not distributed uniformly across different phases of the football microcycle. Recent GPS-based research in professional football has shown that training-to-match external load ratios vary according to microcycle length and the specific external load variable considered [7]. Longer microcycles are generally associated with higher accumulated training-to-match load ratios, whereas shorter microcycles tend to prioritize recovery and tactical preparation [8]. Importantly, high-speed running and sprint-related variables may remain relatively underrepresented during training compared with match demands, whereas acceleration-related loads may be relatively overrepresented [7]. These findings highlight that the quantity and characteristics of training exposure may vary substantially according to the competitive schedule and microcycle structure. Although numerous studies have examined the epidemiology of injuries in professional football, relatively few have investigated seasonal variations in injury characteristics and injury-related time-loss [2]. This study aimed to describe the epidemiology of injuries in a professional football team over three consecutive seasons, including injury incidence, anatomical distribution, injury type, seasonal distribution, and injury-related time-loss. In addition to describing overall injury epidemiology, the present study reports seasonal differences in injury characteristics and injury-related time loss.

## 2. Materials and Methods

Study Design

This retrospective descriptive study examined the epidemiological characteristics of injuries within a professional football team over three seasons. The team’s medical records and injury follow-up data were analyzed retrospectively. The study was conducted and reported in accordance with the STROBE-SIIS (Strengthening the Reporting of Observational Studies in Epidemiology—Sports Injury and Illness Surveillance) guidelines [9]. The completed STROBE-SIIS checklist is provided as Supplementary Table S1. The study was not prospectively registered.

*Study Population*

Data were collected retrospectively from the team’s medical records after the completion of all three seasons. Injury data were collected and coded by the team physician according to the IOC injury surveillance classification system. Following ethical approval, the data were extracted and anonymized in June 2026, when complete injury and exposure data from all three seasons were available for retrospective analysis. A total of 96 unique male first-team players were officially registered with a single professional football club competing in Türkiye across the three-season study period (accounting for player transfers, loans, and squad rotations). Injury surveillance covered all registered players. Of these, 78 players sustained at least one time-loss injury and were included in the injury dataset analyzed in this study. Among these injured players, the mean age was 27.0 ± 4.5 years (range: 18–36 years).

During the study period, the team played a total of 146 matches: 112 league matches, 19 domestic cup matches, and 15 preparation (friendly) matches. During the study period, team exposure was estimated following the recommendations of the international consensus statement for football injury surveillance [9]. Match exposure was calculated as 146 matches × 1.5 h × 11 players, yielding 2409 player-hours. Training exposure was estimated using the scheduled number of training sessions during each seasonal period and an average squad size of 26 players. To better reflect seasonal training density, the preparation period was calculated as eight scheduled training sessions per week (two sessions on each of four training days), whereas the competitive periods were estimated at five scheduled training sessions per week. Each training session was assumed to last 1.5 h. This approach quantified training exposure based on session duration and estimated attendance rather than external training-load variables such as high-speed running distance or acceleration/deceleration counts. Individual-level external load monitoring data, including GPS-derived high-speed running distance and acceleration/deceleration measures, were available for the study period but were not incorporated into the present analysis because the primary objective was to examine injury incidence using standardized exposure estimates based on training and match duration.

The seasonal periods were defined based on the team’s competitive calendar: preparation (mid-June to late July), early season (August to September), mid-season (October to February), and late season (March to May). The total estimated training exposure remained unchanged; only its distribution across seasonal periods was adjusted to better reflect the team’s training structure. For players who transferred to, from, or were loaned by the club during the study period, only the period during which they were registered with the club was included in the exposure and injury analyses.

A total of 260 injuries were recorded during the three-season study period. For each injury, the diagnosis, anatomical location, mechanism, injury environment (training or match), season, and time loss were recorded. Injury records were evaluated in accordance with the definition and data collection principles recommended for international football injury research [10]. The anatomical region, injury type, mechanism, and time lost from sport were coded in accordance with the injury surveillance system recommended by the International Olympic Committee (IOC). Anatomical regions and injury diagnoses were classified according to the IOC coding system [9]. Individual player-level exposure data were not systematically recorded.

*Injury Classification*

Anatomical regions were grouped into seven predefined categories: groin–thigh–lower leg, knee, foot–ankle, hip–gluteal, shoulder–upper extremity, lower back–abdomen, and other.

Injury diagnoses were also reclassified into major injury groups: muscle injuries, ligament injuries, tendon injuries, contusions, meniscus/cartilage injuries, and bone injuries. Other injuries included myofascial pain syndromes (*n* = 18), muscle spasms (*n* = 14), clinically suspected tendinopathies (*n* = 12), neuropathic pain (*n* = 9), and unusual or rare injury conditions that could not be assigned to a specific injury-type category according to the IOC injury surveillance classification system (*n* = 21).

*Time-Loss*

**Figure 1:**
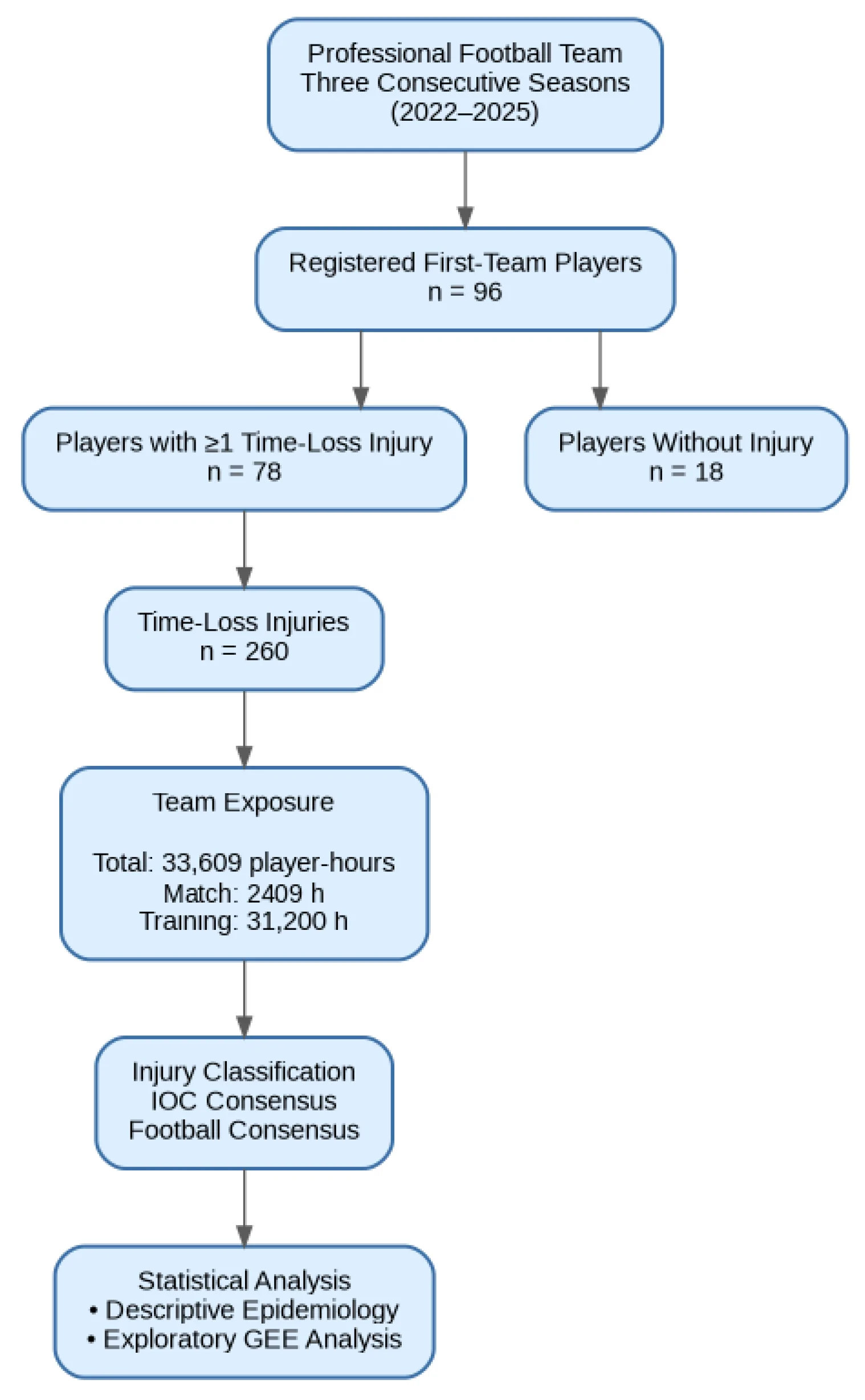
Study Flow Diagram. Flow diagram of player inclusion, injury registration, exposure estimation, and analytical approach used in the study.

Time-loss was defined and recorded as the number of calendar days from the date of injury until the date on which the athlete was deemed medically fit to participate in full team training or competition, according to standard clinical criteria applied by the team physician. Figure 1 provides an overview of the study design, player inclusion, injury registration, exposure estimation, injury classification, and statistical analysis. ChatGPT (OpenAI; GPT-5.6 Luna) was used solely to assist in preparing the initial draft of Figure 1 based on author-provided information and instructions; the final figure was reviewed, edited, and approved by the authors.

## 3. Statistical Analysis

Statistical analyses were performed using IBM SPSS Statistics version 30.0 (IBM Corp., Armonk, NY, USA). Categorical variables were summarized as frequencies (*n*) and percentages (%). No missing data were identified for the variables included in the analyses; therefore, no imputation or other missing-data handling procedures were required. Injury frequencies were estimated as the number of injuries per 1000 player-hours of exposure. As an exploratory secondary analysis, a generalized estimating equation (GEE) model with a Poisson distribution and log link function was used to evaluate differences in injury counts across seasonal periods while accounting for repeated injuries within individual players, as originally described by Liang and Zeger [11]. Player ID was specified as the subject variable and the seasonal period as the within-subject factor, thereby accounting for correlations arising from repeated injury observations within the same player. Because only team-level exposure estimates were available, the natural logarithm of estimated player-hours was included as an approximate offset variable. Therefore, the resulting incidence rate ratios should be interpreted as exploratory estimates rather than precise individual exposure-adjusted injury rates. Results are reported as regression coefficients, with Wald chi-square statistics and corresponding *p*-values. A sensitivity analysis was performed by varying the estimated training exposure by ±10% and ±15%, while keeping match exposure unchanged, to assess the robustness of seasonal injury incidence estimates to plausible variation in training exposure.

## 4. Results

The study evaluated 260 injuries that occurred across three seasons. Of these, 162 (62.3%) occurred during training, and 98 (37.7%) occurred during competition. The general characteristics of injuries recorded during the three-season study period are presented in Table 1.

The higher absolute number of training-related injuries (62.3%) is primarily explained by the substantially greater total exposure time in training (31,200 h) compared with matches (2409 player-hours), despite a lower injury incidence in training (5.2 vs. 40.7 per 1000 player-hours).

A total of 96 unique first-team players were registered across the three seasons. Of these, 78 players (81.3%) sustained a total of 260 time-loss injuries (mean 3.3 injuries per injured player; range, 1–15), whereas 18 players sustained no time-loss injuries. The team competed in the Turkish Super Lig throughout the study period. Eighteen of the 96 registered players did not sustain a time-loss injury during the study period. Consequently, these players did not contribute any injury events to the descriptive injury dataset. All 96 registered players were retained in the exploratory GEE analysis, with players who sustained no injuries contributing zero injury counts across the four seasonal periods. A total of 44 players (56.4%) sustained more than one injury during the three-season study period.

The estimated overall injury incidence was 7.7 per 1000 player-hours. The estimated frequency was 40.7 per 1000 player-hours for match exposure and 5.2 per 1000 player-hours for training exposure. When injuries were stratified by season, 16 (6.2%) occurred in the preparation period, 43 (16.5%) in the early season, 126 (48.5%) in the midseason, and 75 (28.8%) in the late season. The distribution of injuries across seasonal periods is presented in Table 2. When normalized by estimated period-specific exposure, exposure-adjusted injury rates were 2.55, 7.78, 9.29, and 9.12 per 1000 player-hours for the preparation, early-, mid-, and late-season periods, respectively (Table 2). Because these estimates were derived from calendar-based exposure allocation, they should be interpreted descriptively rather than as true calendar-time normalized incidence rates. Sensitivity analysis showed that varying estimated training exposure by ±10% and ±15% did not alter the overall seasonal pattern. Injury incidence remained lowest during the preparation period and highest during the mid- and late-season periods across all exposure scenarios.

The anatomical distribution of injuries is presented in Table 3. The most frequently affected anatomical regions were the thigh (66 injuries, 25.4%), knee (42 injuries, 16.2%), lower leg (39 injuries, 15.0%), groin (32 injuries, 12.3%), and ankle (30 injuries, 11.5%). When injuries were grouped according to the categories defined in the Methods section, the most frequently affected group was the groin-thigh-lower-leg region (137 injuries, 52.7%).

When injuries were evaluated according to the main injury groups, muscle injuries were the most common, accounting for 43.5% (*n* = 113) of total injuries. This was followed by ligament injuries (12.7%; *n* = 33), contusions (6.2%; *n* = 16), tendon injuries (3.8%; *n* = 10), meniscus/cartilage injuries (2.7%; *n* = 7), and bone injuries (2.7%; *n* = 7). Other injuries constituted 28.5% (*n* = 74) of the total injuries (Table 4). The ‘other injuries’ group consisted primarily of non-specific pain complaints, muscle spasms, and various clinical diagnoses that did not fall into the standard categories of muscle, ligament, tendon, or bone injury.

For descriptive purposes, tendon, meniscal/cartilage, and bone injuries were combined into the “Other structural injuries” category in Table 5.

The distribution of injury-related time-loss across seasonal periods is presented in Table 6.

### Exploratory GEE Analysis

The GEE analysis demonstrated a significant association between seasonal period and injury incidence rate (Wald χ^2^ = 41.35, df = 3, *p* < 0.001). The injury incidence rate was significantly lower during the preparation period than during the late season (IRR = 0.17, 95% CI 0.09–0.33, *p* < 0.001), corresponding to an 83% lower estimated injury incidence rate. No significant differences were observed between the early season (IRR = 1.33, 95% CI 0.97–1.83, *p* = 0.080) and the late season, nor between the mid-season (IRR = 1.14, 95% CI 0.90–1.44, *p* = 0.271) and the late season (Table 7).

## 5. Discussion

This study provides a comprehensive description of the injury epidemiology of a professional football team over three consecutive seasons. The principal findings were that injury incidence per 1000 player-hours was approximately 7.8-fold higher during matches than during training, despite the greater absolute number of training injuries; lower-extremity injuries predominated; muscle injuries constituted the largest injury category; and estimated injury incidence was lowest during the preparation period and highest during the competitive season. Overall, these findings are consistent with previous reports describing lower-extremity and muscle injuries as the predominant injury patterns in professional football [2].

Injuries were predominantly located in the lower extremities, with the thigh, knee, lower leg, groin, and ankle accounting for the majority. This pattern closely resembles that reported in recent systematic reviews of professional football injuries [6,12]. Football-specific activities, including sprinting, rapid accelerations and decelerations, changes in direction, and kicking, expose the lower extremities to considerable mechanical stress, providing a plausible explanation for this distribution [2,13]. Groin injuries represented 12.3% of all recorded injuries, a proportion comparable with findings from the UEFA Elite Club Injury Study, in which hip and groin injuries accounted for approximately 14% of all injuries and were predominantly adductor-related [14].

The similarity between our findings and those reported previously supports the view that the repetitive high loads imposed on the adductor muscle group during football-specific movements contribute substantially to groin injury risk. These observations also highlight the importance of reporting both absolute injury counts and exposure-adjusted injury rates, as each metric provides complementary information regarding injury burden.

Muscle injuries accounted for 43.5% of all recorded injuries, confirming that they remain the predominant injury type in professional football. This finding is consistent with previous epidemiological studies, including the UEFA Elite Club Injury Study, which reported that muscle injuries comprise approximately one-third of all injuries, with the hamstring, adductor, quadriceps, and gastrocnemius muscles being most frequently affected [2]. The repeated exposure of these muscle groups to high-speed running, eccentric loading, and explosive football-specific actions may contribute to their high injury incidence. However, the present study did not quantify these external load characteristics at the individual or training-session level. Recent GPS-based research in professional football has shown that high-speed running and sprint-related loads may be relatively underrepresented during training compared with match demands, depending on microcycle length and structure. This training-to-competition load discrepancy may provide a potential framework for interpreting the high injury burden observed in our cohort, although this mechanism could not be directly evaluated in the present study [7]. Similarly, groin injuries represented 12.3% of all recorded injuries in our cohort—a proportion comparable with UEFA findings, in which hip and groin injuries accounted for approximately 14% of all injuries and were predominantly adductor-related [14]. These observations support the view that repetitive high loads imposed on the adductor muscle group during football-specific movements contribute substantially to the risk of both groin and lower-limb muscle injuries. Longitudinal UEFA data have also demonstrated a progressive increase in hamstring injury rates over time [15], reinforcing the importance of implementing evidence-based injury prevention programmes that specifically target muscle injuries.

After adjustment for estimated period-specific exposure, injury incidence was highest during the mid-season (9.29 per 1000 player-hours), similarly high during the late-season (9.12 per 1000 player-hours), and substantially lower during the preparation period (2.55 per 1000 player-hours). Our finding of a lower injury incidence during the preparation period differs from findings reported in male academy football. Veith et al. reported higher medical-attention and non-time-loss injury incidence during the pre-season, while full time-loss injury burden was also higher during the pre-season and end-of-season compared with the start- and mid-season phases [16]. These differences may reflect differences in player age and competitive level, preseason training structure, injury definitions, and exposure characteristics. In contrast, the present study focused exclusively on time-loss injuries in professional first-team players, which may partly explain the different seasonal patterns observed. The high absolute number of injuries observed during the mid-season likely reflects both its longer duration and the cumulative physical demands imposed by prolonged competition. Although accumulated fatigue, fixture congestion, and reduced recovery time have previously been associated with increased injury risk in professional football [15,17], the present data do not permit a direct evaluation of these mechanisms. Recent GPS-based evidence showing that training-to-match external load ratios vary with microcycle length provides a complementary framework for interpreting these seasonal differences, particularly in the context of varying competitive schedules [7]. This framework may be particularly relevant during periods of fixture congestion, when shorter microcycles can alter the distribution of training and match demands. However, fixture congestion and recovery time were not directly quantified in the present study and therefore cannot be considered explanatory factors for the observed seasonal differences. Furthermore, because seasonal exposure estimates were derived from team-level assumptions rather than individual player attendance, these incidence estimates should be interpreted as descriptive, exposure-adjusted estimates rather than precise, individual exposure-based injury rates. The sensitivity analysis indicated that the observed seasonal pattern was robust to moderate variation (±10% and ±15%) in the estimated training exposure. Median injury-related time-loss (3–5 days) remained relatively stable across seasonal periods, suggesting that injury severity did not vary substantially throughout the season. This observation is consistent with previous UEFA findings showing that return-to-play duration is primarily determined by injury type and severity rather than seasonal timing [18].

### 5.1. Limitations

Several methodological considerations should be acknowledged when interpreting these findings. Because individual player attendance records were unavailable, exposure estimates were derived from team-level scheduling assumptions and weighted according to the estimated training density of each seasonal period. Consequently, the exploratory GEE analysis was based on approximate team-level exposure estimates rather than true individual player exposures. In addition, estimated training exposure may have been slightly inflated during periods of higher injury prevalence because injured players were unable to participate in all scheduled training sessions. Such differential exposure misclassification would be expected to produce a conservative bias in incidence estimates by increasing the denominator during periods of greater injury burden. No a priori sample size or statistical power calculation was performed because this was a retrospective descriptive study based on all available injury records from the participating professional football club during the three-season study period. Consequently, the study was not designed to detect predefined differences in injury incidence or injury characteristics, and some exploratory comparisons may have had limited statistical power. Future prospective studies should incorporate an a priori sample size calculation based on predefined primary outcomes and individual-level exposure data. Finally, analyses of injury characteristics and injury-related time loss were descriptive; no formal statistical comparisons of injury severity between seasonal periods were performed. Therefore, these observations should not be interpreted as evidence of seasonal differences in injury severity. Prospective multicentre studies incorporating individual player-level exposure monitoring and larger cohorts are required to confirm these findings and provide more precise estimates of seasonal injury incidence in professional football.

Furthermore, the exposure estimates did not capture the external load content or intensity of individual training sessions, including high-speed running distance and acceleration/deceleration demands. Therefore, the exposure metric reflects time spent in scheduled training rather than the specific physical stimulus experienced by players.

Although individual-level external load monitoring data were available, they were not included in the present analysis. Consequently, the study could not examine whether specific external load characteristics, such as high-speed running or acceleration/deceleration demands, were associated with injury occurrence or seasonal injury patterns.

The relatively large proportion of injuries classified as “other” (28.5%) may also limit the interpretability of injury-type-specific findings because this category comprised heterogeneous clinical presentations. This category included unusual or rare injury conditions that could not be classified into a specific injury-type category according to the IOC injury surveillance classification system. The retrospective nature of the study also precluded a formal assessment of inter-rater or intra-rater reliability of injury coding. However, injury data were coded by the team physician using the standardized IOC injury surveillance classification system.

### 5.2. Strengths

Additional strengths include data collection from three consecutive seasons, a complete review of medical records, consistent injury surveillance within a single professional football club, injury coding according to the internationally accepted IOC injury classification system, and reporting in accordance with the STROBE-SIIS recommendations. The use of the IOC classification system increases the comparability of the findings with other sports injury epidemiology studies. To facilitate interpretation, IOC codes were regrouped into broader anatomical and diagnostic categories based on their clinical similarities.

### 5.3. Clinical Recommendations

Recommendation 1

Continuous implementation of evidence-based muscle injury prevention programmes should be considered throughout the competitive season because muscle injuries were the largest injury category in this cohort. Training strategies that reproduce relevant match-specific demands, including appropriately designed transition games, may also be considered to complement routine injury prevention and help address potential discrepancies between training and competition demands [19].

SORT Strength of Recommendation: C (Based on retrospective descriptive epidemiological data from a single professional football team and complementary external load literature.)

Recommendation 2

Players’ health status and training load should be monitored particularly during the mid- and late-season, as these periods demonstrate the highest exposure-adjusted injury incidence.

SORT Strength of Recommendation: C (Based on retrospective observational data with team-level exposure estimates.)

Recommendation 3

The preseason should be used to optimize physical conditioning, identify previous injuries, and implement individualized injury prevention strategies.

SORT Strength of Recommendation: C (Based on descriptive epidemiological findings and current clinical practice.)

Recommendation 4

Injury surveillance should be maintained throughout the season using standardized injury definitions and exposure recording to facilitate timely identification of injury trends and to support preventive decision-making.

SORT Strength of Recommendation: C (Based on observational findings and international football injury surveillance recommendations.)

Recommendation 5

Quantitative external load monitoring should be incorporated into routine player monitoring, with particular attention to high-speed running distance and sprint-related exposure across training microcycles, to identify potential discrepancies between training and match demands and inform individualized load-management strategies where appropriate.

SORT Strength of Recommendation: C (Based on observational findings and recent external load literature.)

## 6. Conclusions

This descriptive study provides an overview of injury patterns in a professional football team over three seasons. Most injuries occurred during training sessions, primarily involved the lower extremities, and were classified as muscle injuries. When normalized by estimated period-specific exposure, injury incidence was highest during the mid- and late-season periods. Because exposure was estimated at the team level, these findings should be interpreted descriptively. Future confirmatory studies incorporating individual player-level exposure data, larger sample sizes, and analytical methods that account for repeated injuries are needed to provide more precise estimates of injury incidence in professional football.

## Figures and Tables

**Table 1:** General Characteristics of the Study Injuries.

Variable	*n* (%)
Total injuries	260 (100.0)
Training injuries	162 (62.3)
Match injuries	98 (37.7)
Non-contact injuries	162 (62.3)
Contact injuries	83 (31.9)
Overuse injuries	12 (4.6)
Other mechanisms	3 (1.2)
Total matches played	146
Total matches exposure (player-hours)	2409
Total training exposure (player-hours)	31,200
Total exposure (match + training, player-hours)	33,609
Players with ≥1 time-loss injury	78 (81.3)
Players without time-loss injury	18 (18.8)
Total unique players	96

*Training injuries are more frequent in absolute terms due to substantially greater training exposure.*

**Table 2:** Distribution of Injuries, Estimated Exposure, and Injury Rates Across Seasonal Periods.

Seasonal Period	Injuries, *n*	Match Exposure (Player-Hours)	Training Exposure (Player-Hours)	Total Exposure (Player-Hours)	Estimated Injury Incidence (per 1000 Player-Hours)
Preparation Period	16	247.5	6038.7	6286.2	2.55
Early Season	43	495.0	5032.3	5527.3	7.78
Mid-Season	126	990.0	12,580.6	13,570.6	9.29
Late Season	75	676.5	7548.4	8224.9	9.12
Total	260	2409.0	31,200.0	33,609.0	7.74

*Exposure values were estimated based on the team’s competitive calendar and the number of scheduled training sessions for each seasonal period. Therefore, injury frequencies should be interpreted as estimated team-level incidence rates rather than exact individual calendar-time–normalized rates.*

**Table 3:** Anatomical Distribution of Injuries.

Anatomical Region	*n* (%)
Thigh	66 (25.4%)
Knee	42 (16.2%)
Lower leg	39 (15.0%)
Groin	32 (12.3%)
Ankle	30 (11.5%)
Foot and Toe	12 (4.6%)
Abdomen	11 (4.2%)
Shoulder/Clavicle	8 (3.1%)
Gluteal region	7 (2.7%)
Hip	4 (1.5%)
Others	9 (3.5%)
Total	260 (100.0%)

**Table 4:** Distribution of Major Injury Categories.

Injury Category	*n* (%)
Muscle injuries	113 (43.5)
Ligament injuries	33 (12.7)
Contusions	16 (6.2)
Tendon injuries	10 (3.8)
Meniscal/cartilage injuries	7 (2.7)
Bone injuries	7 (2.7)
Other injuries *	74 (28.5)
Total	260 (100.0)

When the distribution of injury groups across seasons was examined, 8 muscle injuries were recorded during the preparation period, 22 during the early season period, 57 during the mid-season period, and 26 during the late season period. * Other injuries included myofascial pain syndromes (n = 18), muscle spasms (n = 14), clinically suspected tendinopathies (n = 12), neuropathic pain (n = 9), and unusual or rare injury conditions that could not be assigned to a specific injury-type category according to the IOC injury surveillance classification system (n = 21).

**Table 5:** Distribution of Major Injury Categories Across Seasonal Periods.

Injury Category	Preparation (*n* = 16)	Early Season (*n* = 43)	Mid-Season (*n* = 126)	Late Season (*n* = 75)	Total (*n* = 260)
Muscle	8	22	57	26	113
Ligament	1	6	15	11	33
Contusion	0	2	12	2	16
Other structural injuries †	3	4	9	8	24
Other (Non-specific injuries)	4	9	33	28	74

*† Other structural injuries include tendon, meniscal, cartilage, and bone injuries.*

**Table 6:** Distribution of Injury-Related Time-Loss by Seasonal Periods.

Seasonal Period	Median (Q1–Q3), Days
Preparation Period	3 (1 to 9)
Early Season	5 (2 to 11)
Mid-Season	4 (1 to 8)
Late Season	5 (2 to 11)

*Median injury-related time loss was 3 days (IQR = 8) during the preparation period, 5 days (IQR = 9) during the early-season, 4 days (IQR = 7) during the mid-season, and 5 days (IQR = 9) during the late-season.*

**Table 7:** Exploratory GEE Analysis of Seasonal Injury Counts.

Variable	B	SE	Wald	*p*	IRR	95% CI
Preparation	−1.764	0.330	28.607	<0.001	0.171	0.090–0.327
Early	0.284	0.162	3.073	0.080	1.329	0.967–1.825
Mid	0.131	0.119	1.213	0.271	1.140	0.903–1.440

Abbreviations: IRR, incidence rate ratio; CI, confidence interval. *Late season served as the reference category.*

## Data Availability

The data supporting the findings of this study are available from the corresponding author upon reasonable request. The data are not publicly available because they were obtained from a professional football club and are subject to privacy, confidentiality, and institutional restrictions. Season-aggregated data are presented in Table 2, Table 3, Table 4, Table 5, Table 6 and Table 7 of the manuscript.

## Supplementary Materials

The following supporting information can be downloaded at: https://www.mdpi.com/article/10.3390/jfmk11030333/s1.

## Abbreviations

IRR	Incidence rate ratio
CI	Confidence interval
