# Supplementary material for: Injury Incidence in Professional Football Is Highest During Mid- and Late-Season: A Three-Season Retrospective Cohort Study
Source: J Funct Morphol Kinesiol. 2026 Aug 25;11(3):333. doi: 10.3390/jfmk11030333 (PMC13608448; doi:10.3390/jfmk11030333)
Supplement: Supplementary file 1 [file jfmk-11-00333-s001.zip › jfmk-4515180-supplementary.pdf]

Supplementary Table S1. STROBE-SIIS Checklist

Study-specific reporting checklist for the three-season retrospective cohort study of time-loss injuries in professional football

Basis: STROBE-SIIS Statement 1.0 (Bahr et al., 2020). The checklist combines the relevant STROBE reporting items with sports-injury/illness-specific extensions.

Item	Reporting requirement (condensed)	STROBE-SIIS extension (condensed)	Location in manuscript	Status
1. Title and Abstract	State design and provide a balanced abstract.	Include sport, athlete population (sex, age, geographic region), competition level, and observation duration.	Title; Abstract	Addressed
2. Background/Rationale	Explain scientific background and rationale.	No additional SIIS-specific requirement.	Introduction	Addressed
3. Objectives	State specific objectives/hypotheses.	State registration information if applicable and the specific surveillance purpose.	Introduction; Methods	Addressed; no registration applicable
4. Study Design	Present key design elements early.	Specify health problems, data-recording approach/tools, coding system, and relevant athlete/sport categorization.	Methods – Study Design; Injury Classification; Figure 1	Addressed
5. Setting	Describe setting, location, dates, exposure/follow-up and data collection.	State location, competition level, surveillance dates, multi-season handling, and prospective/retrospective nature.	Methods – Study Population; Exposure; Data Collection, pp. 4–6	ADDRESSED
6. Participants	Give eligibility, selection/source and follow-up.	Define athlete population and how athletes were selected/recruited.	Methods – Study Population, pp. 4–5	ADDRESSED
7. Variables	Define outcomes, exposures, predictors/confounders/effect modifiers and criteria.	Justify outcome definitions and describe identification of injury outcomes.	Methods – Injury Definition; Exposure; Outcomes	Addressed
8. Data Sources/Measurement	Describe data sources and measurement methods.	Describe surveillance/data collection, coding/classification, sport relationship and exposure measurement.	Methods – Data Collection; Injury Classification; Exposure Estimation	Addressed
9. Bias	Describe efforts to address potential sources of bias.	Report validation/reliability where available and acknowledge data-collection biases.	Methods; Discussion – Limitations	Addressed
10. Study Size	Explain how study size was determined.	No additional SIIS-specific requirement.	Discussion – Limitations, p. 17	ADDRESSED — no a priori sample-size/power calculation was performed; the dataset comprised all available injury records from the three-season period.
11. Quantitative Variables	Explain handling/categorization of quantitative variables.	Explain multiple injuries and injury-severity calculation.	Methods – Injury Definition; Injury Severity; Statistics	Addressed
12. Statistical Methods	Describe statistical methods, subgroup/interaction analyses, missing data, follow-up and sensitivity analyses as applicable.	Specify exposure adjustment, incidence calculation, burden, multiple injuries, athletes not included at outset, and longitudinal follow-up/censoring.	Methods – Statistical Analysis; Sensitivity Analysis	ADDRESSED
13. Participants – Results	Report participant numbers at each stage and reasons for nonparticipation where relevant.	Report athletes followed, affected athletes, injury counts, multi-season counts and effects of transfers/removals.	Results; Figure 1	Addressed
14. Descriptive Data	Report participant characteristics, exposures/confounders, missing data and follow-up time as applicable.	Include competition level and relevant athlete characteristics.	Results; Tables	ADDRESSED
15. Outcome Data	Report outcome events or summary measures over time.	Distinguish injuries from affected athletes and report descriptive injury data appropriately.	Results – Injury Characteristics; Tables 2–7	Addressed

16. Main Results	Report estimates and precision, including adjusted estimates where applicable.	Report exposure-adjusted incidence/prevalence with appropriate CIs and relevant onset/mechanism information.	Results; Tables 2–7	Addressed
17. Other Analyses	Report subgroup, interaction and sensitivity analyses.	Report injury diagnosis information, including region and tissue type, in tables.	Results – Seasonal/Sensitivity Analyses; Tables	Addressed
18. Key Results	Summarize key results in relation to objectives.	No additional SIIS-specific requirement.	Discussion; Conclusion	Addressed
19. Limitations	Discuss limitations, bias/imprecision and direction/magnitude where possible.	Discuss limitations in data collection/coding and risk measures.	Discussion – Limitations, pp. 17–18	ADDRESSED — limitations include exposure estimation, data limitations, heterogeneous injury classification, and absence of an a priori power calculation.
20. Interpretation	Provide cautious interpretation considering objectives, limitations, analyses, similar studies and evidence.	No additional SIIS-specific requirement.	Discussion; Conclusion	Addressed
21. Generalizability	Discuss external validity.	Discuss applicability of athlete population and injury subgroups to broader athlete groups.	Discussion – Strengths and Limitations	Addressed
22. Funding	State funding source and funder role, if applicable.	No additional SIIS-specific requirement.	Funding Statement, p. 21	ADDRESSED — no external funding was received.
23. Ethics	Report relevant ethical considerations.	Describe athlete data privacy/confidentiality in accordance with the Declaration of Helsinki.	Institutional Review Board Statement; Informed Consent Statement; Data Availability Statement, p. 21	ADDRESSED — ethics approval/waiver and privacy/confidentiality measures are reported.

Reference: Bahr R, Clarsen B, Derman W, et al. International Olympic Committee consensus statement: methods for recording and reporting of epidemiological data on injury and illness in sport 2020 (including STROBE Extension for Sport Injury and Illness Surveillance [STROBE-SIIS]). *Orthop. J. Sports Med.* **2020**, *8*, 2325967120902908. https://doi.org/10.1177/2325967120902908.
